# Retrospective Review of the Drop in Observer Detection Performance Over Time in Lesion-enriched Experimental Studies

**DOI:** 10.1007/s10278-014-9717-9

**Published:** 2014-07-09

**Authors:** Sian Taylor-Phillips, Markus C. Elze, Elizabeth A. Krupinski, Kathryn Dennick, Alastair G. Gale, Aileen Clarke, Claudia Mello-Thoms

**Affiliations:** 1 Warwick Medical School, University of Warwick, Coventry, CV4 7AL UK; 2 Department of Statistics, University of Warwick, Coventry, CV4 7AL UK; 3 University of Arizona, Tucson, AZ 85724 USA; 4 Florence Nightingale School of Nursing and Midwifery, Kings College London, London, SE1 8WA UK; 5 Department of Computer Science, Loughborough University, Loughborough, LE11 3TU UK; 6 University of Sydney, Sydney, NSW 2150 Australia

**Keywords:** Observer performance evaluation, Fatigue, Radiologist, Vigilance, Prevalence

## Abstract

The vigilance decrement describes a decrease in sensitivity or increase in specificity with time on task. It has been observed in a variety of repetitive visual tasks, but little is known about these patterns in radiologists. We investigated whether there is systematic variation in performance over the course of a radiology reading session. We re-analyzed data from six previous lesion-enriched radiology studies. Studies featured 8–22 participants assessing 27–100 cases (including mammograms, chest CT, chest x-ray, and bone x-ray) in a reading session. Changes in performance and speed as the reading session progressed were analyzed using mixed effects models. Time taken per case decreased 9–23 % as the reading session progressed (*p* < 0.005 for every study). There was a sensitivity decrease or specificity increase over the course of reading 100 chest x-rays (*p* = 0.005), 60 bone fracture x-rays (*p* = 0.03), and 100 chest CT scans (*p* < 0.0001). This effect was not found in the shorter mammography sessions with 27 or 50 cases. We found evidence supporting the hypothesis that behavior and performance may change over the course of reading an enriched test set. Further research is required to ascertain whether this effect is present in radiological practice.

## Introduction

The pattern of radiologic performance over the course of a workday has previously been investigated [1, 2], but there is little published research on whether or not radiology performance varies over the course of a single reading session (circa 10–100 cases depending on modality/exam). There is evidence of a vigilance decrement developing over the course of a session in fields similar to radiology. A vigilance decrement is a decline in sensitivity to detect targets with time on task, and was first observed over a 30 min session in World War II radar operators [3]. This decline is steeper in highly demanding tasks such as those which have a high event rate, a high working memory load, and low signal salience [4]. Event rate refers to occurrence of background stimulus events in which the critical signals are embedded. Signal salience refers to the conspicuity of the signal in relation to the background noise. Therefore, as many radiologic screening exams are viewed in quick succession, and contain several regions of interest which are difficult to classify as signal or noise, the radiologic screening task may have both low signal salience and a high perceived event rate for the reader.

A second effect which may cause changes to radiological performance with time on task is the prevalence effect. The prevalence effect theory states that performance is dependent on the proportion of actually positive cases in a test set (the level of enrichment or “prevalence”). This effect develops over the course of a reading session because the readers’ expectations change with their experience of the case set. For example, in airport baggage screening when the prevalence is decreased from 50 to 1 % [5], from 98 to 50 % [6], or sinusoidally from 90 to 1 % [6], there is a decrease in sensitivity and an increase in specificity. There is also some evidence for a concurrent increase in performance (d’) [7]. However, Kundel [8] suggested that the prevalence effect is likely to be due to a threshold shift, with any performance changes simply due to differing variances of the distributions of scores applied to normal and abnormal cases. In radiology, the prevalence effect has been observed in detecting pulmonary emboli in pulmonary arteriograms [9] and detecting lung nodules in chest x-rays [8]. However, one study [10] found no prevalence effect in detecting a range of abnormalities in chest x-rays with 2–28 % prevalence.

Wolfe et al. [6] provide a theoretical explanation for the prevalence effect based on the multiple decision model in abnormality detection tasks. In this model, the observer selects targets within the image, evaluates them in comparison to an internal threshold, and will either report based on that target, or continue searching the image for other targets until they reach their “search-quit threshold”, beyond which they estimate further search is not worthwhile. The actual prevalence in the test set affects the observer’s expectations of probability of abnormality for each case. For example, if the first ten cases are all normal one might expect the next ten cases to be mostly normal as well. This decreasing expectation of abnormality at low prevalence increases the observer’s decision threshold for reporting each target, and decreases the search time considered necessary. This theory predicts that performance changes as the reading session progresses. This theory is supported by three observations. Firstly, that interspersing low prevalence tasks with short high prevalence periods “cures” the prevalence effect [7]. Secondly, that in one study of naïve observers in a baggage screening task, the false negative (miss) rate increased as the session progressed in the low prevalence (2 %) condition, but not in the high prevalence condition [7]. Finally, that with decreasing prevalence time decreases for images judged as negative [6].

In radiology, there are few reports of how performance may change over the course of a reading session. Cowley and Gale [11] found that experienced mammography readers reading an enriched test set of 120 cases (approximately one-third malignant) showed a trend towards increasing false negative rates and decreasing false positive (false alarm) rates over the session. Gale et al. [12] describe a slight drop in sensitivity over time reading a test set of 100 symptomatic mammograms, but do not quantify the magnitude of this effect or provide any statistical analysis. This pattern of decreasing sensitivity is consistent with a vigilance decrement, and such a threshold shift is similar to that seen at low prevalence.

In the present research, we investigated whether participant behavior and performance vary with time while examining an enriched test set of radiology cases, including chest and bone x-rays, mammograms, and chest CT scans. The aim was to examine whether the number of cases and reading session length influence results in enriched test set studies and whether there is potential for reading session length to affect radiology performance in practice. This paper re-analyzes the results of six published studies to determine whether performance changed as the reading session progressed [13–18] and is a continuation of our preliminary research on this subject [19]. These six studies were chosen because they are the studies for which we had access to the raw data and complete study protocols beyond what is usually available for meta-analyses.

## Materials and Methods

Ethical approval for this re-analysis of existing data was granted by the University of Warwick’s Biomedical Research Ethics Sub-Committee on 21 March 2011.

Six studies, combined into five datasets, were analyzed. The studies were performed in either the USA or the UK and varied in methodology and research objective. For the analysis, mixed effects models were fitted separately for each dataset.

To standardize terminology, we call cases that are positive on reference standard testing and which feature the condition of interest (such as malignancies, bone fractures, or pulmonary nodules) “abnormal”, and cases that do not feature the condition of interest “normal”. All studies included in this analysis compared two conditions (e.g., time of day, use of film or digital previous mammograms). As the difference between these two conditions is not of primary interest here, we will refer to these simply as “condition 1” and “condition 2”. All studies employed only qualified readers with suitable experience in reading the type of image considered in that study. However, in most studies, there was a subgroup of more experienced senior radiologists who we will refer to as “experienced readers”.

### Studies and Datasets

The Krupinski (2007) study [15] analyzed the influence of 8-bit vs. 11-bit digital displays on radiologists’ performance. One hundred direct digital radiography chest images, half with subtle solitary pulmonary nodules, were read by 18 readers at three US sites, once on the 8-bit and once on the 11-bit display. The readers were 3 residents, 4 fellows, and 11 radiologists, all with at least 1 year of relevant experience.

The Mello-Thoms studies [16, 17] investigated the visual search strategy of readers reading mammograms and the effects of initial mistakes on reading performance. Due to methodological similarities, they could be combined into a single dataset. One hundred three two-view (cranial-caudal and medial-lateral oblique) digital mammograms were read by eight experienced US radiologists. The cases were split into two sessions containing approximately 50 cases each. Forty-four cases had biopsy-proven benign masses, 29 contained biopsy-proven malignant masses, and 27 were lesion-free. The role of visual search strategy and the effects of initial mistakes were assessed using eye tracking equipment.

The Krupinski (2010) study [13] investigated the effect of fatigue in the detection of bone fractures. Sixty musculoskeletal x-rays, half with fractures, were read by 20 radiology residents and 20 radiologists. All cases were read in a single session in which 30 easier cases were presented first and then 30 harder cases were presented. The effect of fatigue was assessed by comparing performance early in the day before any clinical reading and late after a day of clinical reading.

The Taylor-Phillips study [18] investigated the use of prior (film) mammograms in the transition to digital mammography. One hundred sixty cases (94 difficult non-malignant cases and 66 malignant cases) were read by eight experienced UK mammography readers (four radiologists and four radiography advanced practitioners) in sessions of 27 cases. The impact of providing prior mammograms was assessed by comparing reader performance with and without the prior mammograms in separate sessions at least a month apart.

The Krupinski (2012) study [14] analyzed the effect of fatigue in the detection of pulmonary nodules. One hundred sets of chest CTs, each with 20 sections and half with nodules, were read by 22 radiology residents and 22 radiologists. The cases were displayed at a fixed scrolling speed. All 100 cases were read in a single session. The effect of fatigue was assessed by comparing performance early in the work day before any clinical reading and late in the work day after doing clinical reading.

The characteristics of the studies included are detailed in Table 1.

**Table 1:** Characteristics of studies included in the analysis

Study	Participants	Cases	Reading session length	Performance measure	Task	Prevalence of abnormality (%)	Treatment in original investigation
Krupinski et al. 2007 [15]	18	100	100	Confidence of positive decisions (1 = nodule absent, definite, 6 = nodule present definite)	Pulmonary nodule detection in chest x-rays	50	8- or 11-bit displays
Mello-Thoms et al. 2008, 2009 [16, 17]	8	50	50	Confidence of positive decisions 1 to 3 (negative decisions treated as 0)	Detection of abnormal screening mammograms	76	Eye tracking study
Krupinski et al. 2010 [13]	20	60	60	Confidence of positive decisions 0 to 100 % in 10 % intervals (negative decisions treated as 0 %)	Detection of bone fracture on x-rays (all body areas)	50	Before or after a workday
Taylor-Phillips et al. 2012 [18]	8	162	27	Probability of malignancy from 0 % definitely not malignant to 100 % definitely malignant (negative decisions treated as 0 %)	Detection of abnormal screening mammograms	41	Digital mammography with digitized, film, or no previous mammograms
Krupinski et al. 2012 [14]	22	100	100	Confidence of positive decisions 0 to 100 % (negative decisions treated as 0 %)	Detecting lung nodules on CT scans	50	Before or after a workday

Prevalence refers to proportion of cases which contained the abnormality observers were searching for in the task

### Statistical Methods

The studies provided readers with different scales to assess the cases. Some studies used a percentage scale between 0 % (definitely normal/healthy/non-malignant) and 100 % (definitely abnormal/unhealthy/malignant), others asked readers to assign a score between 1 (normal) and 6 (abnormal), or to assign scores between 1 and 3 to only those cases they considered abnormal. To standardize these different scales, the results were reduced to a binary “diagnostic” decision. We considered 50 % or higher for the percentage scales and three or higher for the 1–6 score scales as a verdict for abnormal/unhealthy and any lower value as normal.

For an initial visualization of the datasets, groups of 25 cases were created and mean confidence scores were calculated for each group and dataset. A breakdown of scores for each group of 25 cases was then calculated.

The five datasets varied in number of readers (8–22), number of cases (50–162), number of cases read in a single session (27–100), methodology, objective, and randomization. Thus, a meta-analysis combining study results was not considered appropriate. Mixed effects models were fitted separately for each study dataset. Variable selection for the models was undertaken using a mixed direction forward-selection backward-elimination stepwise procedure. If an interaction term was included, both underlying variables were automatically included in the model. Only significant (*p* < 0.05) covariates were considered for the final model. Relaxing the significance criterion for the variable selection slightly did not yield different final models.

The outcome variables were whether the participant correctly classified the case (using the binary decision described above) and the time taken for classification. Data on time taken was not available for the Krupinski (2007) study. For the Mello-Thoms studies, eye tracking data was available and dwell time was used as an additional outcome. Predictors considered for inclusion in the final model were number of cases since the beginning of the session, whether the case contained an abnormality, reader experience, “condition 1” versus “condition 2”, and interactions. To compensate for a change in case difficulty after 30 cases in the Krupinski (2010) study, a fixed effect for case difficulty was added for this study. Random effects for readers were added for all models and for the Krupinski (2010) study, where reading order was randomized for each reader, a random effect for case ID was added as well. All studies expect Krupinski (2010) used limited randomization of the case order with several different orderings used to simplify the study protocol.

All analyses were performed using the R language for statistical computing, version 2.14.1 [20]. For hypothesis tests, *p* values of less than 0.05 were considered significant. For each study, linear mixed models were fitted using the lme4 package for R, version 0.999902344-0 [21]. Since the outcome variable was binary for the models of performance (correct or incorrect), a logit link function was used for the mixed models. Except when comparing nested models, a restricted maximum likelihood (REML) approach was used.

Confidence intervals and *p* values for linear mixed models (time taken for classification and dwell time) were calculated using Markov chain Monte Carlo (MCMC) provided by the languageR package for R, version 1.4. As MCMC for generalized linear mixed models (correct classification) was not available, approximate *p* values based on Wald Z-tests and approximate confidence intervals based on standard errors were used instead.

The models were validated and the influence of outliers was assessed by repeatedly resampling 95 % of the data, fitting the models and observing the changes in the fit and the resulting *p* values. No notable changes in the fit or *p* values were observed and all outliers were kept in the analysis.

## Results

### Descriptive Statistics

**Fig. 1:**
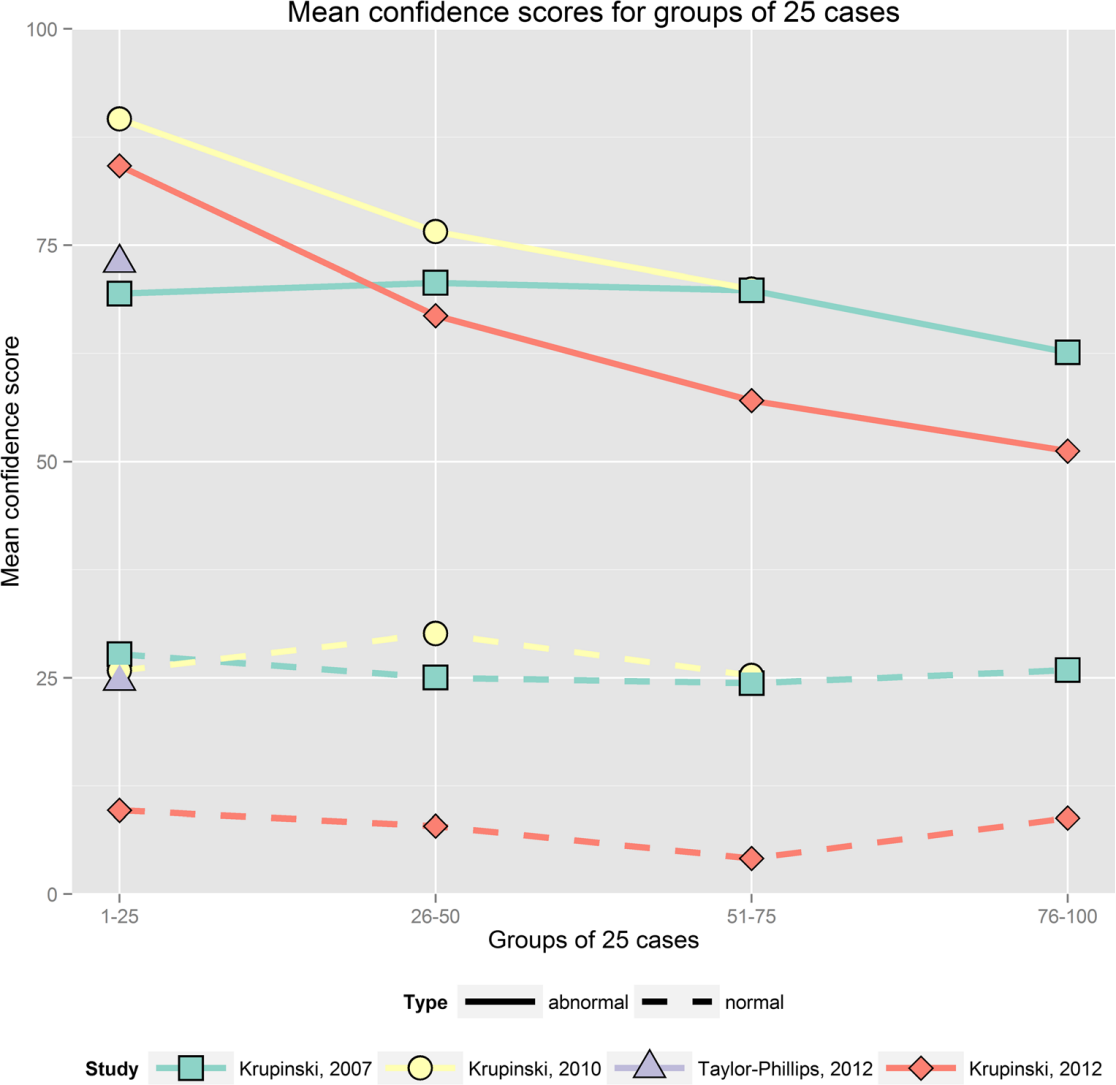
Mean scores assigned to normal (*dashed lines*) and abnormal cases (*solid lines*) as the reading session progressed. To provide a convenient visualization, the cases are grouped into batches of 25 cases and mean scores are calculated for each of those groups. The first case group includes the first 25 cases read, with subsequent groups including subsequent cases in reading order. For Krupinski 2010 [11], the last group includes fewer than 25 cases. For Krupinski 2007, scores from 1 to 6 have been rescaled onto 0 to 100 % for this graphic. For Taylor-Phillips 2012, scores refer to judgement of probability of malignancy rather than confidence

**Fig. 2:**
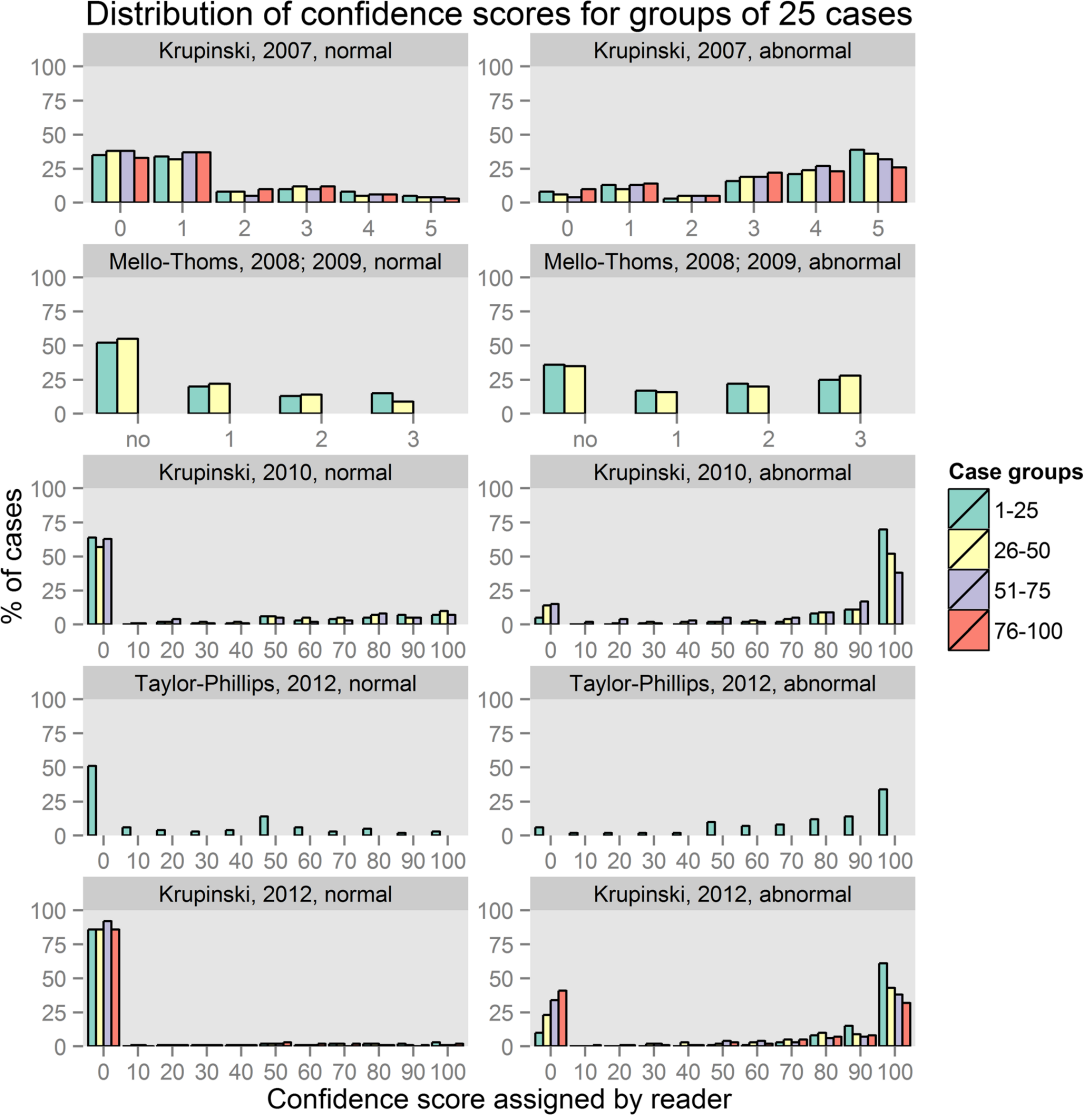
Histograms of scores assigned to normal cases (*left*) and abnormal cases (*right*) in groups of 25 cases in the reading set. The first case group includes the first 25 cases read, with subsequent groups including subsequent cases in reading order. For Taylor-Phillips 2012 and Krupinski 2010, the last group includes fewer than 25 cases. Note that for Taylor-Phillips 2012, scores were rounded to the nearest multiple of 10 to facilitate comparison with the other datasets, and in this dataset scores refer to radiologists judgement of “probability of malignancy” rather than confidence in decision

The confidence scores readers assigned to cases varied considerably between studies. For the three largest studies [1, 14, 15], there is a noticeable reduction in the mean score applied to abnormal cases as the reading session progressed (Fig. 1). This change was driven by a reduction in the number of abnormal cases rated 100 % and/or an increase in the number of abnormal cases rated 0 % as time goes on (Fig. 2).

In the Krupinski (2007) chest x-ray pulmonary nodule study [13], the percentage of abnormal cases that were rated with the maximum score for confidence of abnormality declined from 39 % in the first group of 25 cases to 26 % in the fourth group of 25 cases. In the Krupinski (2010) bone fracture study [11], the percentage of abnormal cases scored highest declined from 70 % in the first 25 cases to 38 % in the last group of cases 51–60. At the same time, the percentage of abnormal cases scored lowest (i.e., definitely no bone fracture) increased from 5 to 15 %. In the Krupinski (2012) CT pulmonary nodule study [12], the percentage of abnormal cases scored highest declined from 61 to 32 % and the percentage of abnormal cases scored lowest increased from 10 to 41 % between the first and fourth groups of 25 cases. This may indicate that any significant decrease in sensitivity over the course of the session would demonstrate a reduction in the number of abnormalities reported rather than a shift in the confidence rating applied to each abnormality. An analysis using mixed models and taking relevant explanatory variables into account was undertaken to provide a more detailed picture of the changes in sensitivity and specificity. In contrast, in the shorter sets the number of abnormal cases scored highest stayed roughly constant at 52–55 % in the Mello-Thoms dataset and 34–37 % in the Taylor-Phillips study.

### Mixed Models for Performance in Individual Studies

**Fig. 3:**
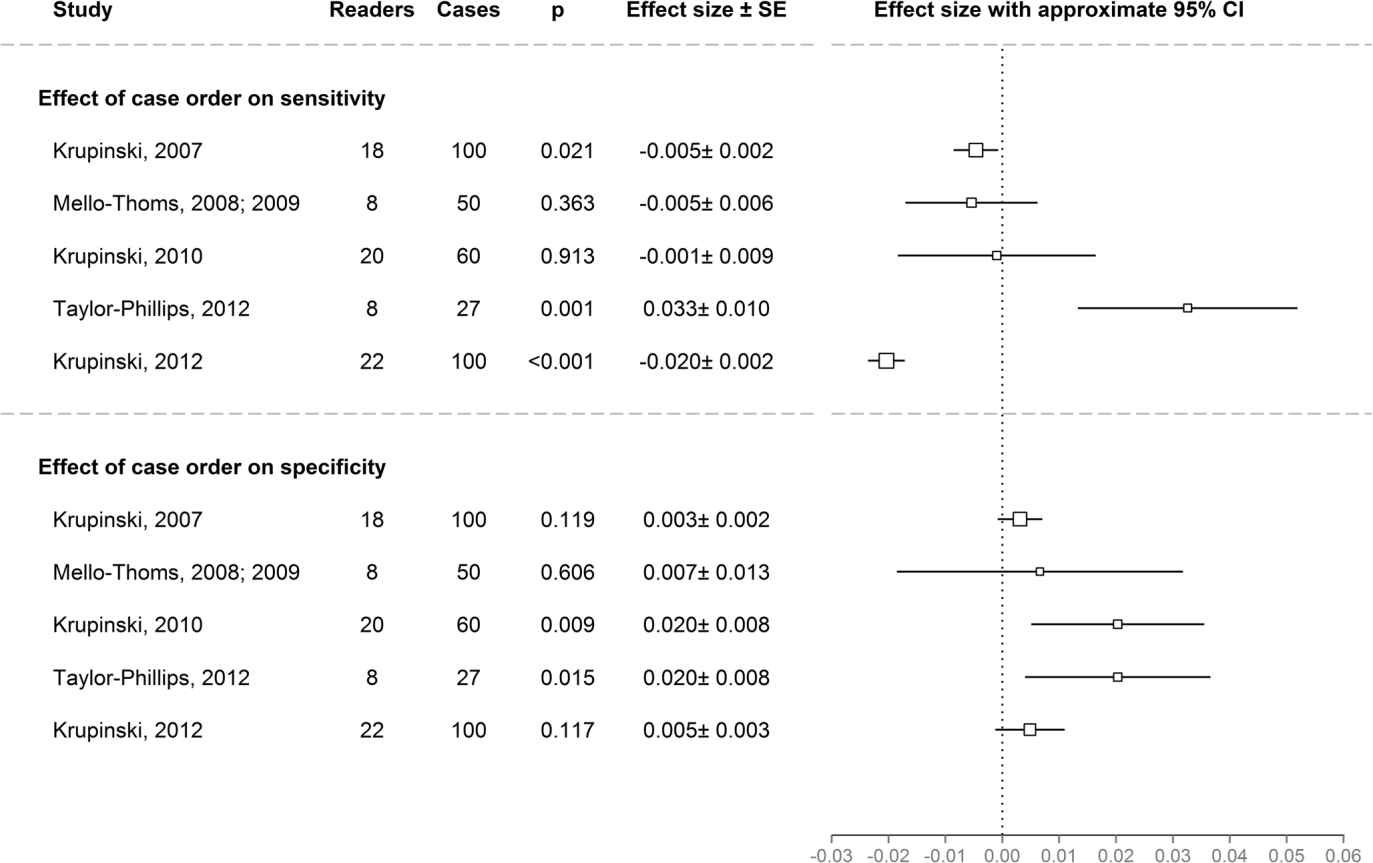
Overview of the effects of case order on sensitivity (*top*) and specificity (*bottom*) cases in the five datasets. Effect sizes were calculated using logit mixed effects models and exp(effect size) is the multiplicative change in the probability of being correct when moving from case i to case i + 1. Positive effects (greater than zero) indicate sensitivity/specificity increasing with time on task. Negative effects (less than zero) indicate sensitivity/specificity decreasing with time on task. The *black box* indicates the estimate for the effect size. The area of the black box is proportional to the standard error. The *lines around the black box* show 1.96 × standard error. *p* values are based on Wald Z-tests. Note that the effects of experience were not considered for this comparison, but the effect for the group of easier cases was considered for the Krupinski 2010 dataset

Any decrease in confidence ratings or increase in threshold as predicted by the vigilance decrement and prevalence effect theories would be demonstrated in the mixed models if the interaction between time (first, second, third etc. case read in that session) and ground truth (whether the case is normal or abnormal) is a significant predictor of a correct decision at 50 % threshold. This interaction was a significant predictor in the three studies with the longest sessions (*p* = 0.0001 to *p* = 0.03). This manifested itself as a significant increase in specificity for two studies (Fig. 3, top) and a significant decrease in sensitivity for two studies (Fig. 3, bottom). However, there was no such interaction in the models of shorter sessions reading 27 or 50 women’s mammograms, and in fact a significant increase in sensitivity was found when reading 27 women’s mammograms [16] (Fig. 3).

In the Krupinski 2007 study [13], sensitivity decreased by eight percentage points (*p* = 0.021) and specificity increased by five percentage points (not significant, *p* = 0.119) over the course of examining 100 chest x-rays. Reader experience was an additional significant predictor, with experienced readers making more correct decisions (*p* = 0.047).

In the Mello-Thoms dataset [14, 15], sensitivity decreased by seven percentage points (not significant, *p* = 0.4) and specificity increased by seven percentage points (not significant, *p* = 0.6), over the course of examining 50 women’s mammograms. In this case set, abnormal (malignant) cases were significantly harder to correctly identify (*p* < 0.001) than the normal (benign or lesion-free) cases. No significant effect for reader experience was found.

In the Krupinski 2010 study [11], sensitivity decreased by one percentage point (not significant, *p* = 0.9) and specificity increased by 27 percentage points (*p* = 0.009) over the course of examining 60 x-rays for bone fractures. There was a slight additional improvement with time for experienced readers (*p* = 0.014). The study featured a set of 30 easier and 30 harder cases, which was accounted for by adding it as a predictor in the model (*p* < 0.001). In this study, the abnormal (bone fracture) cases were significantly easier to correctly identify than normal (no bone fracture) cases, since only difficult “normal” cases were included in this study (*p* < 0.001).

In the Taylor-Phillips study [16], sensitivity increased by 15 percentage points (*p* < 0.001) and specificity increased by 8 percentage points (*p* = 0.015), over the course of examining 27 women’s mammograms. The case set contained abnormal (malignant) cases that were significantly harder to correctly diagnose than normal (benign or normal) cases (*p* = 0.032).

In the Krupinski 2012 study [12], sensitivity decreased by 41 percentage points (*p* < 0.001) and specificity increased by 3 percentage points (not significant, *p* = 0.1), over the course of examining 100 CT scans for lung nodules. In this test set, abnormal (pulmonary nodule) cases were significantly harder to correctly identify than normal (no pulmonary nodule) cases (*p* = 0.003). No significant effect for reader experience was found.

### Patterns in Behavior

**Fig. 4:**
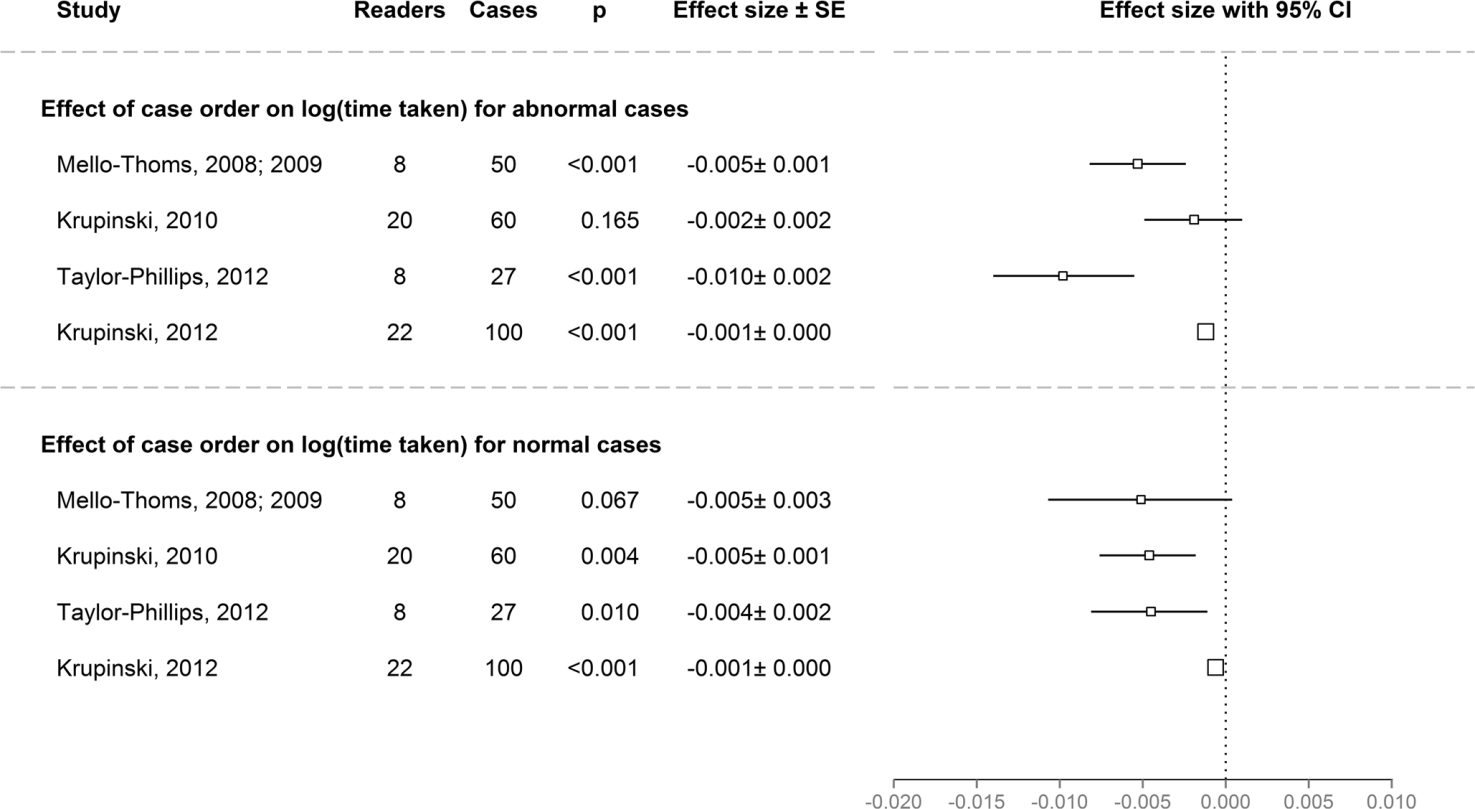
Overview of the effects of case order on time taken for cases classified as abnormal (*top*) and normal (*bottom*) in the five datasets. Effect sizes were calculated using linear mixed effects models and exp(effect size) is the multiplicative change in the log(time taken) when moving from case i to case i + 1. The decrease in time taken per case as the session progressed was significant in all studies. The *black box* indicates the estimate for the effect size. The area of the black box is proportional to the standard error. The *lines around the black box* show 95 % MCMC confidence intervals. *p* values are also based on MCMC. One study (Krupinski 2007) [15] did not have records of time taken per case so could not be included in this analysis

The time taken to examine each case decreased as the reading session progressed in all studies except Krupinski (2007), where time information was not available. This occurred for cases classified as normal as well as abnormal. Over the course of examining the test set, time taken per case, over all subgroups, reduced by 16 % when examining 27 women’s mammograms (*p* < 0.001), by 18 % when examining 60 x-rays for bone fractures (*p* = 0.004), by 9 % when searching 100 CT scans for pulmonary nodules (*p* < 0.001), and by 23 % when examining 50 women’s mammograms (*p* < 0.001) (Fig. 4).

The eye tracking data (visual search) reading 50 mammograms [16, 17] showed that median dwell time was different between response types (*p* < 0.001) and was 1.8 s on true positive locations, 2.3 s on false positive locations and 1.6 s on correct abnormality locations before making a false negative decision. The dwell time decreased as the reading session progressed with an average decrease of 0.6 s between beginning and end of a reading session (*p* < 0.001). This decrease over time did not differ with response type (false negative, true positive, and false positive decisions). Time to first hit, which corresponds to how long radiologists first took to fixate on an abnormality did not change significantly over the course of the reading session (*p* = 0.8).

## Discussion

We re-analyzed data from six previously published enriched case set studies to examine whether reader behavior and performance changed as the reading session progressed. In particular, we were looking for a decline in sensitivity as described by the vigilance decrement and/or a decrease in time taken per case, alongside a sensitivity decrease/specificity increase which would fit with Wolfe’s description of the prevalence effect [6]. In all four datasets which contained time information, we found the time taken per case decreased by between 9 and 23 % as the reading session progressed (all *p* < 0.005), including when reading mammograms, fracture x-rays, and CT examinations. In the three studies with the longest reading sessions, we found evidence of a sensitivity decrease or specificity increase over the course of reading 100 chest x-rays (*p* = 0.005), 60 bone fracture x-rays (*p* = 0.03), and 100 chest CT scans (*p* < 0.0001, interaction between ground truth and time is a significant predictor of correct decisions at 50 % cut-off). In two out of five datasets, this manifested itself as a reduction in sensitivity [14, 15] and in two out of five datasets as an increase in specificity [13, 18]. These differences may be driven by differences in case mix. In the studies with shorter sets of cases (27–50 mammograms), this effect was not seen. In fact, when reading sets of 27 women’s mammograms [18] there was an increase in sensitivity as the reading session progressed, which we did not expect. The largest effect was seen when examining 100 chest CT scans, which had the largest case set with each case having the greatest number of images (multiple slice), and the time per case was limited in the experimental design, which are the conditions known to increase the vigilance decrement [4].

In this research, several published studies were re-analyzed to look for changes in performance and behavior over time. Studies were taken from mammography, chest x-ray, bone x-ray, and chest CT scan from the UK and the USA to establish whether there were consistent patterns in human performance in a range of settings. However, none of these studies were specifically designed to answer the research question, so the design of each was not optimized for this analysis. Therefore, there may be unmeasured sources of systematic bias, and the results should be interpreted with caution. Additionally, studies were not selected by systematic review, and so there may be some bias in the studies selected for inclusion. Due to the exploratory nature of this work, multiple testing corrections were not performed and so resulting *p* values should be considered carefully.

There is little previous research in radiology investigating behavior and performance changes over the course of a reading session. One study [11] showed a trend towards a similar pattern of increasing specificity and decreasing sensitivity over the course of reading 120 mammography cases, but the statistical analysis only showed that performance is not the same at all time-points. Rigorous research measuring changes in baggage screening performance over time by novices [6, 7] demonstrated a threshold shift upwards over time, the magnitude of which was dependent on the prevalence in the case set. In the three studies with longest reading sessions, we found an effect consistent with the findings of Cowley and Gale [11] and Wolfe [6]. However, this effect was not present in sets of 27 or 50 mammograms, and this may be due to the shorter session length in those studies.

Alternatively, the data presented herein may suggest that sensitivity increases at the beginning of a reading session (as shown in the Taylor-Phillips study with 27 cases per session), then plateaus at a certain level (resulting in no significance differences in the Mello-Thoms 2008, 2009 studies, where sessions lasted 50 cases), and finally begins to decline as more cases are read (as shown by the Krupinski 2010 and 2012 studies whose sessions lasted 100 cases), all while specificity increases, no matter how long the reading session is. This behavior may suggest a perceptual priming effect [22], in which earlier in the session radiologists are fine tuned to find cancers, but as the session progresses and few cancers are actually found, the priming effect is reduced, which would be reflected in a lower sensitivity rate at the end of a long reading session. Conversely, priming would also make them better at deciding that cancer is not present, which would result in an increase in specificity as the session progresses.

The theories and observations of both the vigilance decrement and the prevalence effect predict a larger change over time at lower prevalence. If the results observed here are due to these effects, then we would expect to see larger effects in radiology clinical practice. However, if the effects observed here are due to reader adaptation to the test set or experimental conditions in some way, then these effects will not translate into clinical practice. Further research is needed to determine if these effects do impact clinical reading as it would impact patient care.

In this analysis, we found evidence of behavior and performance changes over the course of a reading session. This merits further investigation in a well-controlled environment with a larger participant cohort and optimal randomization to systematically measure how performance and eye tracking behavior change over time at different levels of prevalence and with radiologist experience. Perhaps more importantly, radiologists’ performance over time in clinical practice should be analyzed to determine whether changes over time predicted by the vigilance decrement and the prevalence effect manifest themselves in practice.

## Conclusions

Over the course of a reading session, behavior and performance may change systematically. Further research is required to ascertain whether this effect is present in radiology clinical practice.
